# The Ongoing Journey of a *Shigella* Bioconjugate Vaccine

**DOI:** 10.3390/vaccines10020212

**Published:** 2022-01-29

**Authors:** Patricia Martin, Cristina Alaimo

**Affiliations:** LimmaTech Biologics AG, 8952 Schlieren, Switzerland

**Keywords:** *Shigella*, vaccines, O-antigen, bioconjugate

## Abstract

Shigellosis is a serious disease with a major impact, especially in low-income countries where mortality and morbidity are high. In addition, shigellosis among travelers and military personnel is a cause of significant morbidity and contributes to the increase in antimicrobial resistance. The World Health Organization (WHO) considers the development of a *Shigella* vaccine a priority for public health. Over the past 60 years, several efforts to develop a *Shigella* vaccine have been pursued, without success. The principle of preventing shigellosis with a conjugate vaccine was demonstrated in the 1990′s, but this vaccine was not further developed. Bioconjugation is an innovative technology that allows the production of conjugate vaccines in a biological environment to preserve native immunogenic structures. In this review, we describe the journey of the bioconjugate *Shigella* vaccine, one of the most advanced clinical programs for a *Shigella* vaccine.

## 1. Introduction

Shigellosis is caused by infection with Gram-negative *Shigella* spp. bacteria, a genus belonging to the Enterobacteriaceae family and encompassing four species: *S. dysenteriae*, *S. boydii*, *S. flexneri* and *S. sonnei*. In particular, the flexneri and sonnei species have been identified by the GEMS study [1] as the most prevalent species (65.9% and 23.7% of *Shigella* case isolates, respectively), with five flexneri serotypes accounting for 89.4% of *S. flexneri* cases, namely serotypes 2a, 6, 3a, 2b and 1b [2].

Shigellosis has been reported by WHO as the leading bacterial cause of diarrhoea, accounting for approximately 212,000 deaths—about 13% of all deaths due to diarrhoea [3]. Such clinical outcomes follow the disruption of the intestinal epithelium as a result of invasion by *Shigella* microorganisms, which are able to survive to macrophage phagocytosis, induce macrophage death and disseminate to adjacent cells [4].

Shigellosis is estimated to lead to around 188 million infections annually, including 62.3 million cases in children younger than 5 years [5]. This disease is one of the major causes of morbidity and mortality [6] due to diarrhoea not only among children in low-income countries, but also in travellers and deployed military personnel visiting regions of endemicity. Good hygiene practices, clean water and sanitation are important to reduce transmission of the disease. However, the technical difficulties in implementing such measures, as well as the low infectious dose, make it very challenging to control bacterial spread in areas with poor sanitation. The standard treatment for shigellosis focuses on oral rehydration and antibiotic therapy. However, *Shigella* spp. have acquired resistance to many antibiotics making treatment more difficult and expensive [7]. Though higher for travellers and children or infants in shigellosis-hyperendemic regions, the risk of shigellosis is also increasing in industrialized countries. Outbreaks of shigellosis are increasingly reported, and resistance has increased markedly since 2013. As inexpensive oral antibiotics such as ampicillin and trimethoprim become less effective, alternative drugs such as ciprofloxacin and azithromycin are being used routinely to treat infections. Still, about 77,000 *Shigella* infections in the United States are reported every year to be resistant to such treatments [8], and accordingly, the CDC (Center for Disease Control and Prevention) considers antibiotic-resistant *Shigella* a serious threat. Similarly, the incidence of antibiotic-resistant *Shigella* strains has been increasing in LMICs, with pooled values of multidrug-resistant strains generally above 50% for both *S. flexneri* and *S. sonnei* across Asia, Africa and South America, and above 70% for resistance to ampicillin and tetracycline, or tetracycline and sulphamethoxazole trimethoprim/cotrimoxazole [9].

As initial treatment can fail, resistant infections can last longer than infections with susceptible bacteria, with consequently higher costs for the healthcare system. Therefore, prevention of shigellosis by a vaccine will have an impact well beyond disease and death, and will also slow the spread of antibiotic resistance. Ultimately, as humans are the primary reservoir for *Shigella*, an affordable and effective vaccine could potentially lead to eradication of shigellosis [10]. In line with these thoughts, the WHO’s Product Development for Vaccines Advisory Committee (PDVAC) has identified development of a *Shigella* vaccine as a priority for LMICs and an important goal for public health [3].

Although several *Shigella* vaccine candidates are in development, no licensed vaccine is available yet. Multiple strategies have been followed, including orally administered, live-attenuated [11,12,13] or killed *Shigella* vaccines [14], and recombinant *Shigella* vaccines administered parenterally [15,16,17,18]. Most of these candidates have so far reached phase 1 or 2 in clinical development, providing data on their safety and immunogenicity, and a few have also been tested for efficacy in human challenge models of *Shigella* infection [19,20,21] or in field trials [10,22,23]. To the best of our knowledge, the most advanced ongoing clinical programs are those with the recombinant and parenterally administered candidates; the monovalent *S. flexneri* 2a synthetic conjugate SF2a-TT15 (NCT04602975), previously proven to be safe and immunogenic in healthy adults [15]; the GMMA multivalent candidate altSonflex1-2-3 (NCT05073003), whose monovalent *S. sonnei* precursor was shown to be safe and immunogenic in healthy adults but failed to demonstrate vaccine efficacy in the sonnei challenge model [17,20,24]; the multivalent bioconjugate S4V (NCT04056117), for which safety, immunogenicity and efficacy were shown with its *S. flexneri* 2a component [19,25]. These three candidates are all currently being tested in paediatric populations in Kenya. In addition, a phase 3 study in China with a bivalent *S. flexneri*-*S. sonnei* conjugate was just announced (NCT05156528), and a phase 1 with a live, oral, combined *Shigella*-ETEC vaccine candidate (NCT04634513) and a phase 2 challenge trial with an oral live-attenuated *S. sonnei* vaccine candidate (NCT04242264) will start soon.

In this review, we describe the clinical development of the *Shigella* bioconjugate, one of the most advanced *Shigella* vaccine candidates currently in development.

## 2. O-Antigen-Based *Shigella* Vaccines: From Chemical to Bioconjugates

A glycoconjugate is a hybrid molecule composed of a carrier protein and multiple polysaccharide chains, wherein the polysaccharides are covalently linked to the protein. This linkage of the antigenic polysaccharide to a carrier protein has brought significant advances in the field of vaccinology, eliciting a T-cell-dependent response characterized by the induction of immunological memory and improved immunogenicity [26,27]. This is particularly relevant for some populations at high risk of developing disease, such as children, elderly or immunocompromised individuals, where polysaccharides alone generally fail to mount an adequate and persistent immune response [28,29,30,31]. Indeed, glycoconjugate vaccines have been shown to be safe and effective for different pathogens (i.e., *Neisseria meningitidis*, *Streptococcus pneumonia*, *Haemophilus influenzae*) for more than 30 years [32,33,34].

**Figure 1:**
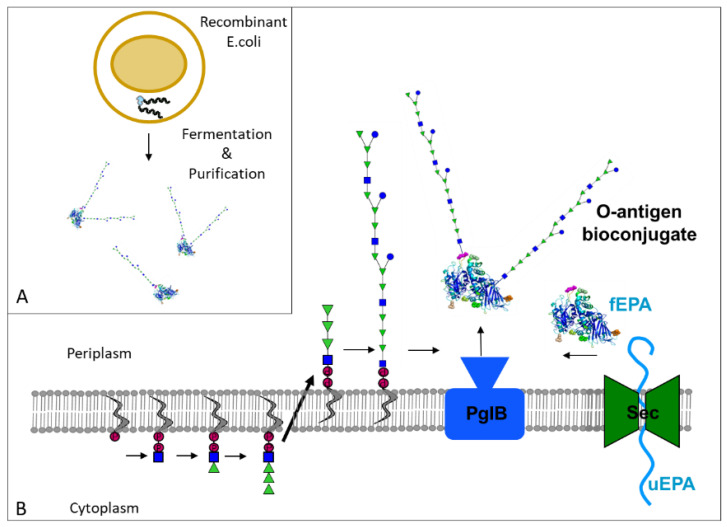
(**A**) Bioconjugates are purified following fermentation and osmotic shock of recombinant *E.coli* cells expressing the soluble glycoproteins in the periplasm. (**B**) Key steps of the in vivo assembly of an O-antigen bioconjugate; O-antigen repeating units are assembled in the cytoplasm on undecaprenol (grey s-shaped line)-pyrophosphate (pink circles with P) lipids, transferred into the periplasm and then polymerized; in parallel, unfolded carrier protein (uEPA) is transferred to the periplasm by the SEC translocation pore (green); in the periplasm, EPA folds (fEPA) and the oligosaccharyltransferase PglB transfers the O-antigen polysaccharide from the undecaprenol pyrophosphate donor to consensus sequences present in the carrier protein EPA, forming a covalent bond. The example illustrates a bioconjugate with the *Shigella flexneri* 2a O antigen. The O antigen is represented using the monosaccharide nomenclature of the Consortium for Functional Glycomics (http://www.functionalglycomics.org/static/consortium/Nomenclature.shtml (accessed on 17 December 2021)).

Bioconjugates are glycoconjugates produced in vivo in bacterial cells (Figure 1). At the core of the bioconjugation is PglB, a bacterial enzyme capable of forming a specific linkage between a glycan and defined sites on a protein moiety whilst preserving the conformation of all antigenic components [35,36]. Using recombinant DNA technologies, the *Escherichia coli* glycan biosynthesis machinery is genetically modified to produce the target polysaccharides and transfer them to acceptor proteins. In the bioconjugation process, the glycoconjugate vaccine is produced entirely in *E. coli* in a single-step process, resulting in advantages for process reproducibility and robustness, while decreasing manufacturing cost. Using this technology, several vaccines against different pathogens including *Shigella* (see Table 1) have been developed and tested in clinical trials. Aside from a few bioconjugates against Gram-positive bacteria such as *Staphylococcus aureus* or *S. pneumoniae* [37], the majority of bioconjugates have so far targeted Gram-negative bacteria such as *Shigella*, and in particular the O-antigen expressed on the surface of these microorganisms.

Based on a combination of challenge studies in volunteers, epidemiological surveillance and nonhuman primate studies, the O-antigen on the bacterial cell surface lipopolysaccharide of *Shigella* spp. has been identified as a key antigenic target, and therefore, a focus for vaccine design. Back in the 1990′s, Robbins and coworkers produced conjugate vaccines consisting of purified *Shigella* polysaccharide conjugated to a protein carrier [38]. Three chemically conjugated *Shigella* O-antigen vaccines [39] have been tested in clinical phases; *Shigella* dysenteriae type 1 polysaccharide conjugated to tetanus toxoid and *S. flexneri* 2a and *S. sonnei* polysaccharides both conjugated to recombinant Pseudomonas aeruginosa exoprotein A (EPA). The three conjugates were shown to be safe and immunogenic. Furthermore, the *S. sonnei* conjugate vaccine was shown in a phase 3 trial to be efficacious in Israeli soldiers and children above 3 years of age [22,40,41]. This demonstrated the validity of *Shigella* O antigens as vaccine targets and that parenteral immunization can be efficacious against shigellosis.

However, none of these conjugates were further developed. Following the age-dependent immune response observed in the field trial in Israeli children, with a drop in efficacy below the age of 3, further effort was invested in the *S. sonnei* chemical conjugate, generating different conjugate configurations with lower molecular mass oligosaccharides comprising the LPS core plus a few O-antigen repeating units [42]. When tested preclinically, such *S. sonnei* chemical conjugates proved to be more immunogenic than the precursor; however, no further reports were published on their development. The high complexity of such a chemical conjugation approach, which is likely to result in a difficult and costly GMP manufacturing process, as well as the discrepancy between the length of the conjugated glycan and its bacterial target, could be postulated as reasons why this *S. sonnei* conjugate was not developed further. The *Shigella* bioconjugate belongs to the next generation of such chemical conjugates, with which it shares the structural characteristics of polysaccharide moieties linked to a protein carrier, but at the same time differs because of its unique biosynthetic process, which ultimately results in epitope preservation and conserved glycan composition.

### 2.1. Shigella Dysenteriae Bioconjugate Vaccine

*S. dysenteriae* is not a common cause of endemic shigellosis but rather of epidemic outbreaks, as seen in Central America, Central Africa, and Southeast Asia, or of vicious outbreaks in confined populations, most notably during refugee situations.

As a proof of principle for the technology, the first bioconjugate vaccine evaluated in humans was against *Shigella* dysenteriae [43]. The O-antigen polysaccharide of *S. dysenteriae* type O1 was bioconjugated to a genetically detoxified version of P. aeruginosa exotoxin A (EPA). The vaccine was produced in *E. coli*, purified, and characterized for clinical testing. A phase 1 study in healthy volunteers was conducted in Switzerland [44] with the objective of demonstrating the safety and immunogenicity of the *S. dysenteriae* bioconjugate (called GVXN SD133). The vaccine was used alone or in combination with Alum hydroxide at two different polysaccharide doses, 2 or 10 µg, and administered twice to 40 healthy subjects, on day 0 and day 60. In summary, both doses of this prototype vaccine were well-tolerated and showed an acceptable safety profile, similar to other licensed conjugate vaccines. It elicited statistically significant O1-specific serum IgG and IgA responses compared to baseline in all groups independent of the formulation and dose of the vaccine, with no significant difference observed between groups. The same level of systemic response (in terms of O1-specific serum antibodies) was detected at the end of the trial, about 5 months later (see Table 1). Functionality of the antibodies raised against the protein carrier, EPA, was also demonstrated via in vitro neutralization assay, confirming that the native configuration of the protein carrier is preserved during the bioconjugation process, as has previously been shown for other protein carriers [45].

This first-in-human study paved the way for the bioconjugation technology to move forward with additional vaccine targets [46,47], including a multivalent *Shigella* vaccine with O antigens from *S. sonnei* and from *S. flexneri* 2a, 6 and 3a, to provide broad coverage against the most prevalent *Shigella* O-antigen serotypes [2].

### 2.2. Shigella Flexneri Bioconjugate Vaccine

In the process of developing a multivalent *Shigella* vaccine, and to further demonstrate the robustness of the bioconjugation platform, another *Shigella* bioconjugate vaccine was generated and clinically evaluated. The O antigen from *S. flexneri* 2a was chosen based on its high prevalence in endemic shigellosis. It also offered the possibility to use an existing human challenge model for preliminary evaluation of vaccine efficacy, thus advancing the development of a *Shigella* bioconjugate. The same protein carrier previously used for the O1 bioconjugate, EPA, was selected.

The initial phase I study [16] was conducted in a single centre at the NMRC (Naval Medical Research Center) to evaluate the safety and immunogenicity of the vaccine. Thirty healthy adult volunteers were enrolled in the study and received two injections one month apart of 10 µg polysaccharide of the *S. flexneri* 2a bioconjugate (called Flexyn2a), alone or in combination with an aluminium adjuvant.

Safety and immunogenicity of this second prototype bioconjugate vaccine were confirmed, and similar to the observations in the study with the dysenteriae vaccine, immunization with Flexyn2a elicited a significant increase in IgG and IgA titres against *S. flexneri* 2a LPS (see Table 1). Both vaccine groups, with or without adjuvant, showed a ≥16-fold increase in *S. flexneri* 2a-specific IgG and IgA GMT after the first dose, and overall, ≥92% of the participants seroconverted (≥4-fold increase from baseline). Analysis between groups did not show significant differences between the adjuvanted and nonadjuvanted groups or between first or second injection, although the study was not powered to identify differences. Despite the small sample size analysed, the lack of difference observed could indicate that a single injection without adjuvant is sufficient to generate an immune response in the adult population. Functionality of the vaccine-induced antibody responses was confirmed by serum bactericidal assay (SBA). In addition, *S. flexneri* 2a-specific antibody-secreting plasma cells (IgA and IgG) could be identified following vaccination.

The positive results obtained in this phase 1 trial warranted the further evaluation of the prototype monovalent vaccine Flexyn 2a in the controlled human infection model (CHIM study) to provide a preliminary basis to evaluate vaccine efficacy.

The randomized, double-blinded and placebo-controlled phase 2b trial [19] was conducted at the Johns Hopkins Bloomberg School of Public Health. Sixty-seven participants received two injections of 10 µg of Flexyn2a vaccine or placebo, one month apart. One month after the second dose, 59 participants received a target oral challenge of 1500 CFU (colony-forming units) of *S. flexneri* 2a strain 2457T (see Table 1).

The vaccine showed a good safety profile, similar to what was observed in the phase I study. In terms of immunogenicity, after the first dose the IgG responses already increased around 10-fold compared to baseline. The second vaccination or the subsequent challenge did not further increase the antibody titers of vaccinees, which stayed significantly higher when compared to the level reached in placebo recipients following challenge (2.5-fold increase in IgG titers in placebo recipients). In terms of responders (recipients who had at least a 4-fold increase in IgG), four weeks after the first dose the responder rate was 76.5%, and four weeks after the second dose the rate was 81.8%. As described in more detail in Clarkson et al. [25], several other parameters were investigated in this study, including memory B-cell responses, bactericidal assays and gut-homing LPS-specific antibody responses. All confirmed that the vaccine induced a robust and functional systemic immune response and supported activation of a vaccine-specific response at the mucosal level as well.

To determine the efficacy of the vaccine, the primary definition of established shigellosis included “severe diarrhea” (six or >800 g loose stools within 24 h) or “moderate diarrhea (four to five or 401–800 g loose stools within 24 h) with fever or with one or more moderate constitutional or enteric symptom” or “dysentery” (which included at least two loose stools with gross blood within 24 h and any reportable constitutional symptom). Based on this definition, vaccine efficacy (VE) was 30% (13/30 vs. 18/29; *p* = 0.11; 95% CI: −15 to 62.6). However, vaccination with the bioconjugate was observed to have conferred significant protection against more severe diarrhea (≥10 or ≥1000 g loose stools within 24 h) with a 72% efficacy (2/30 vs. 7/29; *p* = 0.07; 95%CI: −9.5 to 64.3), reduced severity of symptoms and was efficacious toward additional clinical endpoints including antibiotic administration and need for oral rehydration. The efficacy of the bioconjugate against more severe clinical outcomes of shigellosis was then confirmed with a post hoc analysis, whereby more severe shigellosis criteria were considered, such as fever or severe symptoms (rather than moderate or mild symptoms as in the per-protocol primary endpoint) along with diarrhea (at least moderate). The remarkably higher VE, namely 51.7% (8/30 vs. 16/29; *p* = 0.02; 95%CI: 5.3 to 77.9), obtained with the more severe shigellosis definition indicated that the vaccine was indeed efficacious. A shigellosis definition based on more severe outcomes was more appropriate for demonstrating vaccine efficacy in the context of the CHIM, as well as better reflecting the most disabling symptoms for subjects with shigellosis.

As described in Talaat et al., efficacy of the bioconjugate was additionally confirmed by different analyses, including a shigellosis disease score. This was shown to be lower in vaccinees, even those who developed shigellosis, than in placebo recipients, further demonstrating that even if disease was not totally prevented by vaccination, the severity was decreased.

In general, the study showed a clear association between immune responses elicited by vaccination, and protection against shigellosis after oral challenge, with 2a-LPS specific serum IgG responses being the parameter best associated with protection. Lower shedding was also reported for those vaccinees with the highest level of anti Sf2a-LPS IgG and vice versa for vaccinees with a weaker IgG response, suggesting a role for anti-Sf2a LPS serum IgG in limiting bacterial replication in the gut. In terms of duration of response, approximately one-year post-vaccination, the level of anti SF2a LPS-serum IgG in the vaccinated group was still significantly higher than at baseline, and significantly higher compared to challenged placebo recipients. Exploratory analyses performed as part of this study also showed activation of mucosal immunity following parenteral immunization and association between such mucosal responses and protection against disease [48].

### 2.3. Quadrivalent Shigella Bioconjugate Vaccine

The promising results obtained with the Flexyn2a prototype further supported the development of a multivalent bioconjugate vaccine, aiming to provide broad protection against the most prevalent serotypes of *Shigella*. The S4V is a quadrivalent bioconjugate vaccine which carries O-antigens of *Shigella flexneri* serotypes 2a, 3a, 6 and *Shigella sonnei* bioconjugated to the EPA carrier protein. These O antigens were chosen as they are from the most prevalent strains and will enable coverage to reach around 85% [49]. Based on *Shigella* incidence data from the GEMS study [50], such a vaccine may result in a direct coverage of 64%. Additional coverage of approximately 20% toward *Shigella* serotypes not included in the vaccine could be reached because of cross reactivity, as previously shown preclinically with immune sera against 2a and 3a *Shigella* serotypes [2].

A phase 1/2 dose-finding and age-descending (adults–children–infants) double-blind study is currently ongoing in Kenya with S4V to evaluate vaccine safety and immunogenicity in the target population of 9-month-old infants (NCT04056117). The trial has enrolled about 600 participants at two Kenya Medical Research Institute (KEMRI) sites in Kilifi and Kericho. Four different doses with or without aluminum adjuvant are being evaluated with a two-dose vaccination schedule at 9 and 12 months followed by a booster 6 months later. The data collected in this study will be an important step in the development of a *Shigella* vaccine to help protect the most vulnerable populations in low-income countries and will also contribute to the scientific knowledge around immune responses against *Shigella* O antigens in the pediatric population. The study completion is planned for the beginning of 2023, with results expected to be available during 2023. A positive outcome for safety and immunogenicity from this trial will significantly support pivotal efficacy trials with the *Shigella* bioconjugate in the target pediatric population, as well as in travelers and military personnel going to *Shigella* endemic regions.

## 3. Outlook and Conclusions

Although there have been many years of effort, a safe and efficacious vaccine against *Shigella* is not yet available. With the incorporation of molecular diagnostics, the burden of *Shigella* disease is now clearer than ever and a *Shigella* vaccine will have a significant impact in reducing morbidity, mortality and antimicrobial resistance. Although efforts to improve hygiene and appropriate sanitation may help to reduce the incidence of shigellosis, an effective vaccine remains a priority. According to the 2018 Wellcome Trust report “Vaccines to Tackle Drug Resistant Infections”, enteric diseases have a massive impact on the development of antimicrobial resistance due to the quantity of antibiotics used to treat these infections. As reported, experts believe that a vaccine which helps to reduce disease severity, even if it does not prevent disease completely, will be valuable in decreasing the use of antibiotics.

An ideal *Shigella* vaccine needs to be safe and effective in infants, children, and adults, and simple and affordable to manufacture. The bioconjugate *Shigella* vaccine could fulfil all these characteristics. From the mechanisms of action, bioconjugate vaccines are expected to perform the same as other conjugate vaccines. Conjugate vaccines have been demonstrated to be well-tolerated and are routinely used to protect against several childhood diseases. There are several examples of successful conjugates vaccines on the market which generate immunity and long-lasting protection. Therefore, the expectations are that the bioconjugate *Shigella* vaccine will generate an adequate immune response in infants and children. The safety, immunogenicity and efficacy data collected during phase I and 2b (CHIM) studies with Flexyn2a in adults confirmed that the technology is appropriate for the generation of an effective *Shigella* vaccine.

From the manufacturing perspective, bioconjugation technology is very promising and can produce a low-cost vaccine due to the simplicity of the technology and reduced manufacturing steps. By producing the vaccines in a well-established biological system and avoiding chemical steps, bioconjugation technology generates a homogeneous product with low batch variability and few analytical requirements.

In this review, we summarize the development of the bioconjugate *Shigella* vaccine. This is, to our knowledge, the most advanced active program in the *Shigella* field. With the observed efficacy against severe shigellosis outcomes, the phase 2b challenge trial provided clinical proof of concept of efficacy with the monovalent bioconjugate. In addition, it derisked and streamlined the development of S4V, the final multivalent product. In the ongoing clinical trial, it remains to be seen if the quadrivalent bioconjugate vaccine can generate significant immunogenicity above the level of preexisting antibodies in children and in immunologically naïve infants. This being the case, there is hope that this quadrivalent bioconjugate could be efficacious against disease.

Indeed, though results identifying a correlate of protection (CoP) are still considered preliminary, the strong association with protection identified for serum IgG with the Flexyn2a bioconjugate is in line with previous reports on the relevance of serum IgG in protection against shigellosis. In particular, field studies in Israel with the chemical conjugate vaccine for *S. sonnei*, with a 70% efficacy against shigellosis in children >3 years old [22], highlighted the association between anti-LPS serum IgG and protection against shigellosis. Results of the CHIM study with Flexyn2a confirmed these previous findings and highlighted the importance of a strong serum antibody response against *Shigella*-LPS to achieve protection against disease. Although different immunization strategies (oral, parenteral, intranasal, etc.) may ultimately result in multiple CoP (mechanistic or not), at least for parenteral immunization, anti-LPS serum IgG titers are currently supported as a candidate for CoP.

It is important to note that the size of the inoculum administered in a CHIM (significantly higher than what is usually seen in nature) may set an artificially high bar for a vaccine candidate, potentially resulting in lower efficacy in this setting than would be observed for the same vaccine candidate in a field trial. However, human challenge trials generally represent a powerful tool to elucidate the pathogenesis of diseases, identify CoP and accelerate the development of vaccines, as shown for cholera or typhoid vaccines [51,52]. To accelerate the development of the bioconjugate vaccine, one strategy could be the demonstration of efficacy against *Shigella sonnei* and Flexneri 2a, the two serotypes for which a CHIM is established. The S4V quadrivalent vaccine could then be used, accompanied by collection of additional safety, immunogenicity and possibly preliminary field-efficacy data, in the target population of infants. Full confirmation of efficacy could follow in postlicensure phase IV studies [53]. Such a strategy could avoid long and costly phase 3 field studies and ensure earlier delivery of a vaccine highly needed in the pediatric population. At the same time, these results could support licensure of the *Shigella* vaccine for travelers or military. It will be important to work with regulatory agencies to identify any possible nontraditional paths to licensure in order to bring a safe and efficacious vaccine to populations who need it in the shortest time possible.

## Figures and Tables

**Table 1:** Summary of clinical studies conducted with *Shigella* bioconjugates.

Study ID	Bioconjugate	Doses * & schedule	Number (*n*) & Age	Results
NCT01069471 Phase I, Switzerland, in 2010	Vaccine against *S.dysenteriae* (GVXN SD133-EPA)	2 µg PS 2 µg PS + AlOH_3_ 10 µg PS 10 µg PS + AlOH_3_ 2 injections: Day 0 and 2 months	*n* = 40 18–50 years	- No safety concerns - Increase in anti-O1 serum IgG at day 30 vs. baseline - No further/significant anti-O1 serum IgG increase after second dose or with adjuvant - Anti-O1 serum IgG titers maintained for 5 months (last evaluation)
NCT02388009 Phase I, US, in 2015	Vaccine against *S. flexneri*-2a (Flexyn2a-EPA)	10 µg PS 10 µg PS + AlOH_3_ Placebo 2 injections: Day 0 and 1 month	*n* = 30 18–50 years	- No safety concerns - Increase in anti-2a serum IgG, IgA and SBA titers following vaccination - No further/significant anti-2a serum IgG increase after second dose or with adjuvant - Additionally, for the phase IIb: - Positive correlation between immune response and protection against shigellosis - Vaccine efficacy against shigellosis: 30% to 50% (depending on outcome definition) - Vaccine efficacy against more severe diarrhea > 70% - Vaccination reduced incidence and severity of constitutional enteric symptoms - Vaccination reduced shigellosis disease score
NCT02646371 Phase IIb, US, in 2016		10 µg PS Placebo 2 injections: Day 0 and 1 month	*n* = 67 18–50 years	
NCT04056117 Phase I/II, Kenya, Started in 2019, ongoing	Vaccine against *S. flexneri* 2a, 3a, 6 *and sonnei* (S4V-EPA)	- Two Vaccine doses: Medium and High ± AlOH_3_ - 2 injections: day 0 and 1 month - Two Vaccine doses: Medium and High ± AlOH_3_ - 3 injections: day0, 1 and 7 months - Four Vaccine doses: Very Low, Low, Medium and High ± AlOH_3_ - 3 injections: day 0, 3 and 9 months	*n* = 16 18–50 years *n* = 48 2–5 years *n* = 528 9 months	Ongoing, enrollment completed

* doses are indicated as amount of specific O-antigen polysaccharides (PS) conjugated to EPA.

## Data Availability

Not applicable.
